# UBE2QL1 is Disrupted by a Constitutional Translocation Associated with Renal Tumor Predisposition and is a Novel Candidate Renal Tumor Suppressor Gene

**DOI:** 10.1002/humu.22433

**Published:** 2013-10-07

**Authors:** Naomi C Wake, Christopher J Ricketts, Mark R Morris, Elena Prigmore, Susan M Gribble, Anne-Bine Skytte, Michael Brown, Noel Clarke, Rosamonde E Banks, Shirley Hodgson, Andrew S Turnell, Eamonn R Maher, Emma R Woodward

**Affiliations:** 1 Centre for Rare Diseases and Personalised Medicine, School of Clinical and Experimental Medicine, College of Medical and Dental Sciences, University of BirminghamBirmingham, UK; 2 University of Wolverhampton, Wulfruna StreetWolverhampton, UK; 3 The Wellcome Trust Sanger Institute, Wellcome Trust Genome CampusHinxton, Cambridge, UK; 4 Department of Clinical Genetics, Vejle HospitalVejle, Denmark; 5 Genito Urinary Cancer Research Group, School of Cancer and Enabling Sciences, Paterson Institute for Cancer Research, The University of Manchester, Manchester Academic Health Science Centre, The Christie NHS Foundation TrustManchester, UK; 6 Cancer Research UK Clinical Centre, St. James's University HospitalLeeds, UK; 7 South West Thames Regional Genetics Service, St. George's Medical School, University of LondonLondon, UK; 8 School of Cancer Sciences, College of Medical and Dental Sciences, University of BirminghamBirmingham, UK; 9 West Midlands Regional Genetics Service, Birmingham Women's HospitalEdgbaston, Birmingham, UK; 10 Academic Department of Medical Genetics, Addenbrooke's Treatment Centre, Hills RoadCambridge, UK

**Keywords:** renal cell carcinoma, UBE2QL1, ubiquitin conjugating enzyme, FBXW7

## Abstract

Investigation of rare familial forms of renal cell carcinoma (RCC) has led to the identification of genes such as *VHL* and *MET* that are also implicated in the pathogenesis of sporadic RCC. In order to identify a novel candidate renal tumor suppressor gene, we characterized the breakpoints of a constitutional balanced translocation, t(5;19)(p15.3;q12), associated with familial RCC and found that a previously uncharacterized gene *UBE2QL1* was disrupted by the chromosome 5 breakpoint. *UBE2QL1* mRNA expression was downregulated in 78.6% of sporadic RCC and, although no intragenic mutations were detected, gene deletions and promoter region hypermethylation were detected in 17.3% and 20.3%, respectively, of sporadic RCC. Reexpression of *UBE2QL1* in a deficient RCC cell line suppressed anchorage-independent growth. *UBE2QL1* shows homology to the E2 class of ubiquitin conjugating enzymes and we found that (1) UBE2QL1 possesses an active-site cysteine (C88) that is monoubiquitinated in vivo, and (2) UBE2QL1 interacts with FBXW7 (an F box protein providing substrate recognition to the SCF E3 ubiquitin ligase) and facilitates the degradation of the known FBXW7 targets, CCNE1 and mTOR. These findings suggest *UBE2QL1* as a novel candidate renal tumor suppressor gene.

## Introduction

Renal cell carcinoma (RCC) is the most common adult renal tumor and is genetically and histologically heterogeneous [Kovacs et al., 1997; Maher, 2011]. Although only about 3% of RCC are familial, these cases provide a paradigm of how the elucidation of inherited causes of cancer can provide important insights into the molecular mechanisms of sporadic cases. Thus, germline mutations in the *VHL* (MIM #608537) tumor suppressor gene (TSG) cause the familial cancer syndrome von Hippel–Lindau disease (MIM #193300) and somatic *VHL* inactivation is found in at least 80% of sporadic clear cell RCC [Banks et al., 2006; Foster et al., 1994; Gnarra et al., 1994; Latif et al., 1993; Maher et al., 1991]. The identification of the *VHL* gene product, pVHL, and its function as the substrate recognition unit of the E3 ubiquitin ligase complex that regulates the proteolytic degradation of the α-subunits of the hypoxia inducible transcription factors HIF-1 and HIF-2, led to recognition of the fundamental role of hypoxia inducible gene pathway activation in the pathogenesis of familial and sporadic RCC [Cockman et al., 2000; Maxwell et al, 1999]. However, not all RCC is associated with HIF dysregulation and alternate pathways of sporadic RCC tumorigenesis remain to be identified.

Familial RCC is genetically heterogeneous and many patients do not have mutations in known inherited RCC genes [e.g., *VHL*, *FH* (MIM #136850, #150800), *FLCN* (MIM #607273, #135150), *MET* (MIM #164860, #605074), *SDHB* (MIM #185470, #115310, #171300) [Maher, 2011; Ricketts et al., 2008; Woodward et al., 2008]. It has long been recognized that inherited RCC may be associated with constitutional chromosome translocations, particularly of chromosome 3 [Cohen et al., 1979]. To date, 13 RCC-associated translocations have been described and, in some cases, characterization of the breakpoints has led to the identification of candidate TSGs (e.g., *FHIT*, *LSAMP*, *NORE1*) that have been implicated in sporadic renal tumorigenesis [Cohen et al., 1979; Kuiper et al., 2009; Woodward et al., 2010]. Thus, although the association of constitutional chromosome 3 translocations with RCC has been well established [Woodward et al., 2010], the significance of nonchromosome 3 constitutional translocations is more difficult to evaluate. Here, we describe a novel nonchromosome 3 constitutional translocation associated with an inherited predisposition to RCC and investigate the function and role in sporadic renal tumorigenesis of *UBE2QL1*, a previously uncharacterized gene that is disrupted by the translocation.

## Materials and Methods

### Cell Lines and Clinical Samples

An EBV-transformed lymphoblastoid cell line was established from the index case of the family with the t(5;19)(p15.3;q12) by the European Cell and Culture Collection, Porton Down, UK and maintained in RPMI 1640 (Invitrogen, Paisley, UK) supplemented with 10% FCS at 37°C and 5% CO_2_. RCC cell lines KTCL-26, SKRC 45, SKRC 54, Caki-1, 786-0, KTCL-140, RCC4, SKRC 39, SKRC 47, SKRC 18, UM-RC-3, RCC48, RCC1, RCC12, A498, ACHN, 769-P, CAL-54 were routinely maintained in DMEM (Invitrogen, Paisley, UK) supplemented with 10% FCS at 37°C and 5% CO_2_.

### Characterization of t(5;19)(p15.3;q12) Breakpoints

Flow-sorted derivative chromosomes were initially hybridized onto BAC arrays as described [Fiegler et al., 2003], which resolved the breakpoints to within 1 Mb. This information was used to design custom arrays (Roche-Nimblegen, Welwyn Garden City, UK) to further refine the breakpoints, which were then characterized by PCR and Sanger sequencing.

### Immunohistochemistry

Immunohistochemistry was performed using 5 μm tumor sections. Specimens were deparaffinized in xylene and alcohol. Antigen retrieval was achieved by water bathing the slides in low pH target retrieval solution at 98°C and treated with 1% H_2_O_2_ in 98% alcohol to block endogenous peroxidase. Slides were then incubated at room temperature for 1 hr with primary antibody and treated with the EnVision + kit (Dako, Cambridgeshire, UK). Slides were then visualized using diaminobenzidine and counterstained in Mayer's hematoxylin solution (Sigma–Aldrich, Poole, UK).

### *VHL* and *UBE2QL1* Mutation Analysis

DNA was extracted from peripheral leucocytes using the Nucleon BACC2 kit (GE Healthcare [Amersham Biosciences], Buckinghamshire, UK). *UBE2QL1* (NM_001145161.2) and *VHL* (NM_000551.3) were screened for mutations by PCR amplification of all coding exons and exon–intron boundaries followed by a terminator cycle sequencing reaction containing PCR products, BigDye Terminator v3.1 and 5x sequencing buffer (Applied Biosystems). PCR conditions and Primer sequences are available on request. Products were run on an ABI 3730 automated sequencer (Applied Biosystems).

### Quantitative Real-Time PCR

RNA was extracted from renal tissue following a traditional phenol:chloroform RNA extraction technique. cDNA was derived from 1 μg of RNA template using SuperScript® II Reverse Transcriptase and random hexamers following manufacturer's instructions (Invitrogen, Paisley, UK). Taqman quantitative real-time (qRT)-PCR was undertaken using Gene Expression Taqman Assays (20× FAM dye-labeled) for *UBE2QL1* and *β-actin* along with Taqman Universal Master Mix II (10×) (Applied Biosystems). Fifty nanograms of cDNA samples was loaded in triplicate onto a 96-well plate along with negative controls containing no cDNA. *β-actin* probes were used as internal controls and were loaded in triplicate on the same plate for each sample. PCR conditions were used as recommended by Applied Biosystems. Reactions were run on 7500 Real-Time PCR System (Applied Biosystems).

### 5-Aza-2′-Deoxycytidine Treatment

The demethylating agent 5-Aza-2′-deoxycytidine (Sigma–Aldrich, Poole, UK) was freshly prepared in double-distilled H_2_O and filter sterilized. Cell lines were plated in 75 cm^2^ flasks in DMEM supplemented with 10% FCS at differing densities. Twenty-four hours later, cells were treated with 5 μmol/l 5-Aza-2′-deoxycytidine. The medium was changed 24 and 72 hr after treatment. RNA was prepared 5 days after treatment using RNABee (AMS Biotechnology, Abingdon, UK). *UBE2QL1* expression was detected by reverse transcriptase-PCR. Primers and PCR conditions available on request.

### Bisulfite Modification and Methylation Analysis

Bisulfite modification was undertaken using the EpiTect Bisulfite Kit following manufacturer's instructions (QIAGEN, Crawley, UK). The *UBE2QL1* CpG island was identified on the human genome browser (http://genome.ucsc.edu/) and the promoter region amplified from RCC cell lines and primary tumors. Primer sequences are available on request. PCR products were digested with *Bst*UI and visualized on a 2% agarose gel. Bisulfite-modified products selected for sequencing were cloned into the pGem®-T Easy Vector (Promega, Southampton, UK) according to the manufacturer's instructions. Up to 12 individual colonies were chosen for initial amplification followed by sequencing using vector specific primers.

### Multiplex Ligation-Dependent Probe Amplification

Multiplex ligation-dependent probe amplification (MLPA) analysis to detect copy number changes was performed using the SALSA MLPA P300-A1 human DNA reference-2 kit according to manufacturer's instructions (MRC-Holland, Amsterdam, Netherlands). Custom *UBE2QL1* probes were designed using MRC-Holland guidelines (http://www.mlpa.com/WebForms/WebFormMain.aspx) in regions with no known polymorphisms as determined using the SNP database (http://www.ncbi.nlm.nih.gov/SNP/). PCR products were separated using an automated 3730 DNA Analyzer and analyzed using GeneMapper® *ID* v3.1 (Applied Biosystems). Peak heights for each probe were intrasample normalized using the peak heights of reference probes in the same sample. Probes that deviated from adjacent reference probes ≥25% were highlighted. An average deviation of ≥30% from adjacent reference probes for all *UBE2QL1* probes or single exon probes indicated a whole gene or exon deletion respectively.

### Plasmid Constructs

*UBE2QL1* expression constructs were made by cloning the full length human coding region amplified from RCC cell lines into the *Eco*R1–*Bam*H1 sites of pcDNA3.1 (Invitrogen, Paisley, UK), pFLAG-CMV4 (Sigma–Aldrich, Poole, UK), and pCMV-myc (Clontech, Saint-Germain-en-Laye, France) vectors. The FBXW7 coding regions were cloned into p3XFLAG-myc-CMV-24 (Sigma–Aldrich, Poole, UK) at the following sites: FBXW7α in *Hin*d3–*Eco*RI; and FBXW7γ in *Hin*d3–*Xba*1. The FBXW7 constructs were a gift from Markus Welcker and the HIS_6_-ubiquitin vector a gift from Dirk Bohmann. Stop codons were included in the FBXW7 coding regions so the myc tag was not incorporated. The point mutants for *UBE2QL1* were generated by PCR-based site-directed mutagenesis using the QuikChange Lightning Site-Directed Mutagenesis Kit following manufacturer's instructions. Plasmids were verified by sequencing.

### Antibodies

The following antibodies were used: monoclonal anti-FLAG M2 [1:1,000 dilution Western blot (WB), 1:2,000 dilution immunofluorescence (IF); Sigma–Aldrich, Poole, UK]; monoclonal anti-β-actin (1:10,000 dilution WB; Sigma–Aldrich, Poole, UK), monoclonal antitubulin (1:10,000 dilution WB and IF; Sigma–Aldrich, Poole, UK), anti-myc (1:1,000 dilution WB and IF; Sigma–Aldrich, Poole, UK), anti-HIS_6_ (1:1,000 dilution; Abcam, Cambridge, UK), anti-mTOR (1:1,000 dilution; Cell Signaling, Hitchin, UK), anti-CCNE1 [1:1,000 dilution WB, 1:20 dilution immunohistochemistry (IH); Abcam, Cambridge, UK], monoclonal anti-CA9 (1:400 dilution IH; Abcam, Cambridge, UK), monoclonal anti-CCND1 (1:25 dilution IH; NeoMarkers, Fremont, CA).

### Transfection

HEK-293, HeLa, SKRC 39, and SKRC 47 cells were transfected with various plasmids using Fugene Transfection Reagent (Roche) according to the manufacturer's protocol.

### Colony Formation Assays

5 × 10^5^ of SKRC 39 and SKRC 47 cells were transfected with either 2 μg of empty vector or expression vector. Forty-eight hours after transfection cells were seeded in a serial dilution and maintained in 10 ml DMEM with 10% FCS supplemented with 1 mg/ml G418 (Sigma–Aldrich [Life Technologies], Poole, UK). Twenty-one days after initial seeding surviving colonies were stained with 0.4% crystal violet (Sigma–Aldrich, Poole, UK) in 50% methanol. Each transfection was undertaken in triplicate.

### Anchorage-Independent Growth Assays

RCC clones of SKRC 47 stably expressing FLAG-UBE2QL1 or empty vector control were suspended in 2 ml DMEM, 10% FCS, 3% agar. Cells were maintained by addition of 200 μl of DMEM, 10% FCS, supplemented with 1 mg/ml of G418 weekly. Colonies measuring ≥100 μm were counted following 5 weeks of incubation in soft agar. Each experiment was undertaken six times simultaneously.

### Coimmunoprecipitation

Transfected cells were lysed with NETN buffer [50 mM Tris–HCl at pH 7.5, 150 mM NaCl, 1 mM ethylenediaminetetraacetic acid (EDTA), 1% Nonidet P-40] and whole-cell lysates obtained by centrifugation were incubated with 10 μg of specified antibody bound to protein G Dynabeads (Invitrogen, Paisley, UK) for 2 hr at 4°C. The beads were then washed with NETN 4× buffer and applied to SDS-PAGE. Immunoblotting was carried out following standard protocols.

### His Pull-Down Assay

Following transfection, HEK-293 cells were lysed with 1× binding/wash buffer (50 mM Na-phosphate at pH 8.0, 300 mM NaCl, 0.01% Tween-20) containing 1% Triton X-100. The whole-cell lysates obtained by centrifugation were incubated with 2 mg of His Tag Isolation and Pulldown Dynabeads® (Invitrogen, Paisley, UK) for 1 hr at 4°C. The beads were then washed four times with 1× pull-down buffer (3.25 mM Na-phosphate at pH 7.4, 70 mM NaCl, 0.01% Tween-20) and eluted with 50 μl of His elution buffer (300 mM imidazole, 50 mM Na-phosphatase pH 8.0, 300 mM NaCl, 0.01% Tween-20). Laemmli sample buffer was added to the elutes with or without β-mercaptoethanol as indicated and SDS-PAGE undertaken. Immunoblotting was carried out following standard protocols.

### Reticulocyte Lysate Protein Transcription/Translation

Wild-type UBE2QL1 and the UBE2QL1 C88A mutant were synthesized with L-α-[^35^S]-methionine using the pcDNA3.1 constructs as template and the TNT® Coupled Reticulocyte Lysate System following manufacturer's instructions (Promega, Southampton, UK).

### Immunofluorescence

Cells grown on cover-slips were washed three times with PBS then fixed with chilled methanol for 10 min at 4°C. Cells were then washed three times in PBS and blocked with 1% BSA in PBS for a minimum of 30 min. Primary antibody diluted in 1% BSA in PBS was added to the cells and incubated for 1 hr at room temperature. After washing with PBS, cells were incubated with either goat antimouse IgG Alexa Fluor 594 or goat antirabbit IgG Alexa Fluor488 (Invitrogen, Paisley, UK) secondary antibodies at room temperature for 1 hr. After a final wash with PBS, cover-slips were mounted with 4,6-diamidino-2-phenylindole (DAPI) nucleic acid stain (Invitrogen, Paisley, UK). Cells were analyzed using a fluorescence light microscope, Axiovert 200 (Zeiss).

### Degradation Assay

HeLa cells were transfected with either myc-UBE2QL1 or empty vector control. At 24 hr posttransfection, cells were treated with cyclohexamide (Sigma–Aldrich, Poole, UK) at a concentration of 100 μg/ml and collected at the following times afterward: 0; 2; 4; 6; 8; and 10 hr. Whole-cell extracts were prepared by RIPA lysis (50 mM Tris–HCl pH8, 150 mM NaCl, EDTA 1 mM, 0.5% Na-deoxycholate, 1% NP-40, and 0.1% SDS) and centrifugation. Ten micrograms of protein was analyzed by SDS-PAGE and immunoblotting with the indicated antibodies.

### Statistical Analysis

The data are expressed as mean ± SEM from a suitable number of experiments as indicated in the figure legends. Statistical analysis was by the two-tailed Student's *t*-test and *P* < 0.05 was considered significant.

## Results

### Clinical Case Report

A family with an apparent genetic predisposition to RCC was referred to our clinic. The index case presented at the age of 35 years with separate foci of an 18 mm oncocytoma and a 5 mm chromophobe RCC in her left kidney (Supp. Fig. S1). Subsequently, her sister was diagnosed at the age of 36 years with two RCC (a 2.5 cm clear cell and 1.0 cm chromophobe) in her right kidney and, 2 years later, multiple oncocytomas (*n* = 4, 0.6–1.8 cm) in her left kidney. Their deceased mother was reported to have developed a carcinoid tumor at the age of 44 years and their maternal aunt reported to have died from an RCC (age unknown). No mutations were detected in either *VHL* or *FLCN* but a constitutional balanced translocation, t(5;19)(p15.3;q12), was identified in both sisters but not in the two unaffected brothers. We surmised that the constitutional translocation was responsible for the family history of renal tumorigenesis and proceeded to characterize the translocation breakpoints.

### Characterization of t(5;19)(p15.3;q12) Breakpoints

**Figure 1:**
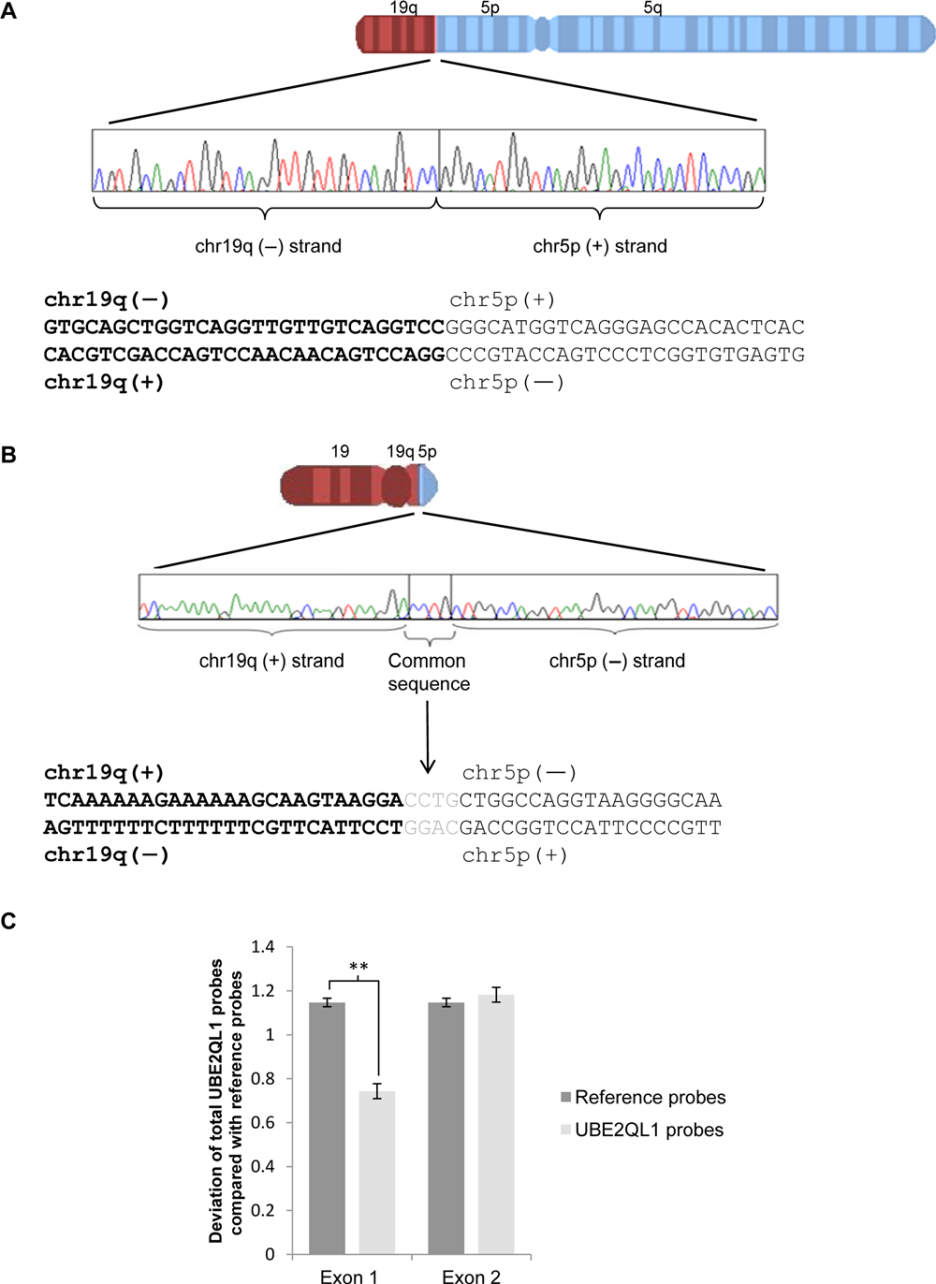
Characterization of t(5;19)(p15.3;q13.1) breakpoints and identification of an intragenic deletion of *UBE2QL1* in a t(5;19)(p15.3;q13.1) associated renal tumor. A: Sequence of der(5) breakpoint. B: Sequence of der(19) breakpoint. There are four bases, CCTG, for which it was not possible to ascribe their chromosomal origin. C: Multiplex ligation-dependent probe amplification bar chart showing deviation of *UBE2QL1* probes from reference probes for exons 1 and 2 in a t(5;19)(p15.3;q13.1) associated renal oncocytoma. There is a significant deviation for exon 1 probes indicating a deletion of exon 1 (unpaired *t*-test, error bars = SEM, ^**^P < 0.01).

We initially mapped the breakpoints by hybridization of amplified flow sorted derivative chromosomes onto the whole genome tiling path array held at the Wellcome Trust Sanger Institute [Fiegler et al., 2003]. The localizations of the breakpoints were further refined using a custom designed oligonucleotide CGH array (Nimblegen, OH) followed by direct genomic sequencing (following long range PCR amplification) across the breakpoints (Supp. Fig. S2A and B; Fig. 1A and B). The chr19 breakpoint was defined at 30,279,438 on der(19) and at 30,279,436 on der(5) and the chr5 breakpoint at 6,456,990 on der(19) and at 6,456,998 on der(5) (hg19). There were four bases, CCTG, present at the der(19) breakpoint which are common to both chr19q and chr5p in the breakpoint region and it was not possible to assign their chromosomal origin. The chr19 breakpoint was associated with a duplication of either GGACCTG (30,279,436–442) or GGA (30,279,436–438) and the chr5 breakpoint with a deletion of either CAGGGCT (6,456,991–997) or GCT (6,456,995–997) depending on the origin of the four common bases. We noted the chr5 breakpoint was found to disrupt the first intron of a previously uncharacterized gene, *UBE2QL1*, so that the 5′ region, exon 1, and part of intron 1 were translocated onto chr19. No gene was disrupted by the chromosome 19 breakpoint. To determine if there was evidence of *UBE2QL1* functioning as a classical TSG (as for *VHL*), we investigated whether allele loss might occur in a renal tumor from the family. As one allele of *UBE2QL1* had been found to be disrupted in the t(5;19)(p15.3;q12) carriers, we investigated whether there was loss of the second allele in the t(5;19)(p15.3;q12) associated renal tumors. We did not find evidence of loss of chr5 but did detect an intragenic deletion of *UBE2QL1* exon 1 in an oncocytoma from patient III:II (Fig. 1C).

### Identification of *UBE2QL1* Genetic and Epigenetic Alterations in Sporadic RCC

**Figure 2:**
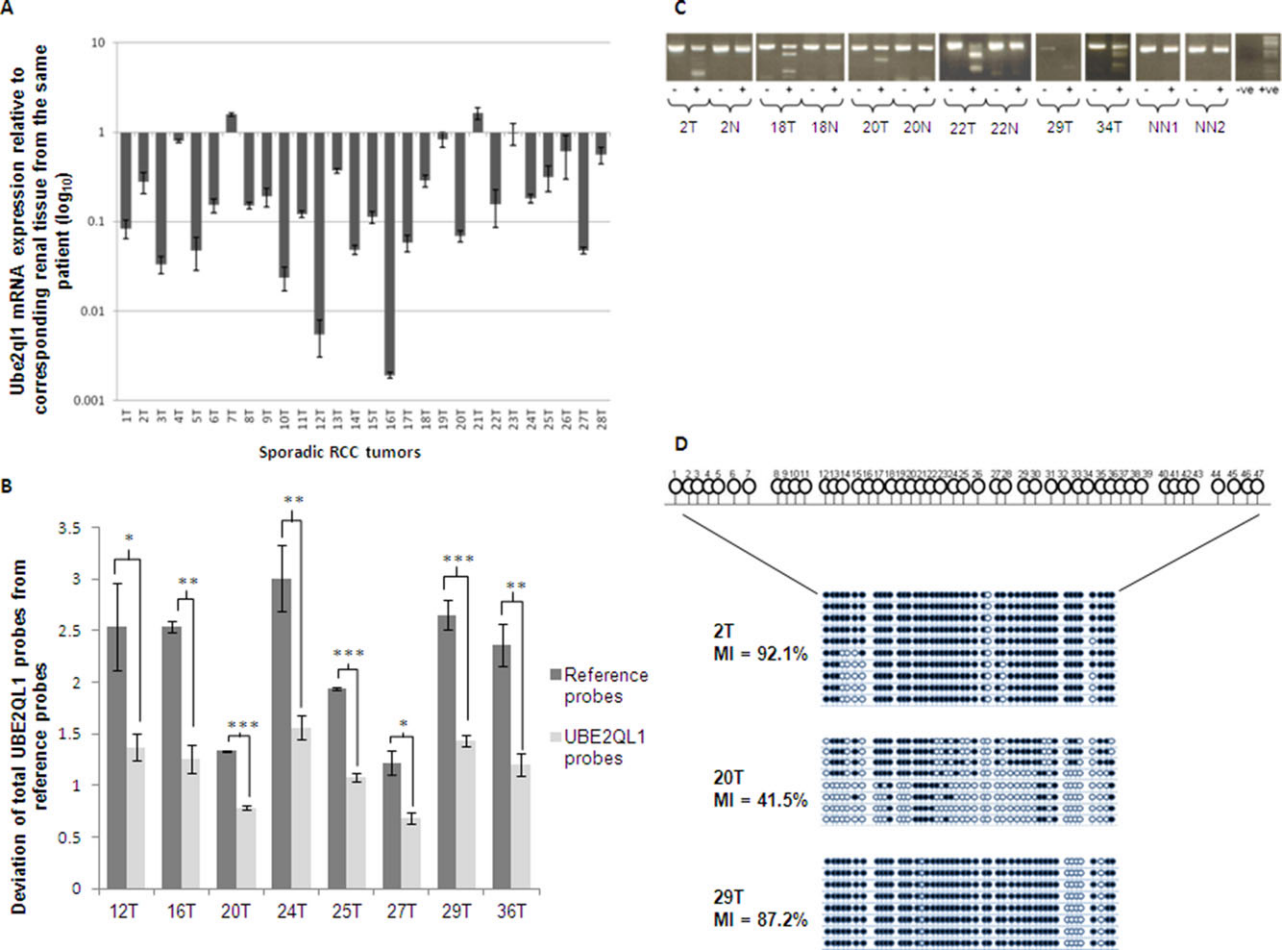
Identification of *UBE2QL1* genetic and epigenetic alterations in sporadic RCC. A: Quantitative real-time PCR bar chart showing % of *UBE2QL1* expression in 28 sporadic RCC compared with corresponding normal renal tissue (*n* = 3). β-Actin was used as an internal control. 22/28 tumors showed a >40% loss of expression. B: Multiplex ligation-dependent probe amplification bar chart showing deviation of all *UBE2QL1* probes from reference probes in eight sporadic RCC. A significant deviation of all the *UBE2QL1* probes from the reference probes indicates a complete gene deletion (unpaired *t*-test, error bars represent SEM, *P < 0.05, ^**^P < 0.01, ^***^P < 0.001). C: BstU1 digest of bisulfite modified DNA (COBRA) for the *UBE2QL1* CpG island in six sporadic RCC. Methylation was detected in the tumor samples (T) shown but not in either corresponding (N) or unrelated (NN) normal renal tissue. “−” no BstU1 added, “+” BstU1 added. “−ve” no DNA template, “+ve” PCDNA3.1 positive control for in vitro methylation. Smaller bands in both “−” and “+” lanes represent primer dimer. D: Schematic diagrams representing sequence analysis of the *UBE2QL1* promoter region following cloning of bisulfate-modified DNA. CpG dinucleotides analyzed are numbered 1–47. Vertical lines represent individual CpGs, black circles represent methylated CpGs, and open circles unmethylated CpGs. The methylation index (MI) is calculated as a percentage of all the methylated CpGs/total number of CpGs sequenced.

As some inherited RCC genes (e.g., *VHL*, *MET*) have also been implicated in the pathogenesis of sporadic RCC, we therefore investigated whether *UBE2QL1* also has a role in sporadic RCC tumorigenesis. Initially, we investigated *UBE2QL1* mRNA expression levels and found loss of expression in 11/18 (61.1%) RCC cell lines (Supp. Fig. S3A) and, compared with corresponding normal tissue, in 22/28 (78.6%) sporadic RCC (Fig. 2A). Although direct sequencing of *UBE2QL1* in 17 RCC cell lines and 116 sporadic RCC did not detect intragenic mutations, analysis for copy number abnormalities using a custom-designed MLPA assay detected heterozygous *UBE2QL1* deletions in 8/49 (16.3%) sporadic RCC (Fig. 2B). The MLPA detected deletions were confirmed by loss of heterozygosity at closely linked microsatellite markers (D5S2505 and D5S2054) in three informative cases (Supp. Fig. S4).

Epigenetic inactivation by promoter region hypermethylation is a common mechanism of TSG inactivation in renal and other cancers. A *UBE2QL1* CpG island was detecting using http://cpgislands.usc.edu/ set to the standard criteria [Takai and Jones, 2002] and treatment with the demethylating agent 5-Aza-2′-deoxycytidine (5-Aza) in the 11 *UBE2QL1* silenced RCC cell induced reexpression of *UBE2QL1* in five (Supp. Fig. S3B). Methylation of the *UBE2QL1* 5′ CpG island (Supp. Fig. S5) was confirmed by COmbined Bisulfite Restriction Analysis (COBRA) in the five RCC cell lines with reduced expression levels, suggesting *UBE2QL1* gene expression is dysregulated by CpG island methylation (Supp. Fig. S3C). We therefore investigated whether *UBE2QL1* promoter hypermethylation was present in primary RCC and we detected hypermethylation in 14/66 (21.2%) primary RCC tested (but not in matched normal kidney where available) (Fig. 2C and D). *UBE2QL1* mRNA expression data were available for eight RCC cases with promoter region hypermethylation and each tumor demonstrated ≥60% reduction in expression compared with matched normal tissue. 2/41 tumors with both COBRA and MLPA analysis showed homozygous inactivation of *UBE2QL1*.

### *UBE2QL1* Suppresses RCC Cell Growth

**Figure 3:**
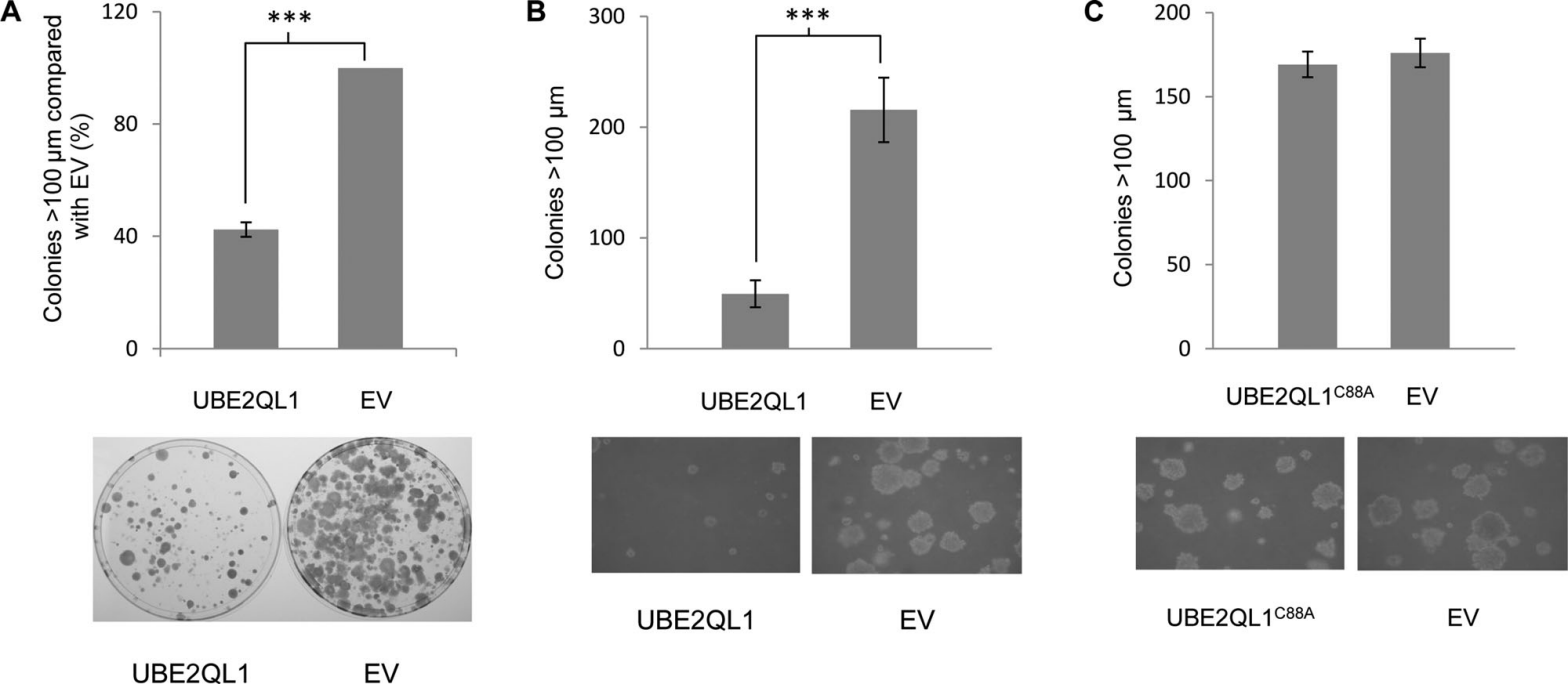
*UBE2QL1* shows growth suppression and inhibition of anchorage-independent growth. A: Colony growth assays with SKRC 47 RCC cell lines stably expressing pFLAG-CMV-4 (EV) or pFLAG-CMV-4-*wt*UBE2QL1 (UBE2QL1). Colonies (>100 μm) were manually counted blindly after 4 weeks (*t*-test, error bars = SEM, ^***^P < 0.0001, *n* = 3). SKRC 47 cells not expressing UBE2QL1 (EV) produced significantly more large (>100 μm) colonies compared with those expressing exogenous UBE2QL1 (UBE2QL1). Below the graph is a representative plate showing colony reduction after UBE2QL1 reexpression. B: Stable clones of SKRC 47 pFLAG-CMV-4-*wt*UBE2QL1 and pFLAG-CMV-4 were seeded at the same density into soft agar and incubated for five weeks. SKRC 47 cells not expressing UBE2QL1 (EV) produced significantly more large (>100 μm) colonies compared with cells expressing exogenous UBE2QL1 (UBE2QL1) (*t*-test, error bars = SEM, ^***^P < 0.0001, *n* = 6). Below the graph are representative images of clones following 5 weeks of incubation (×100 magnification). C: Stable clones of SKRC 47 pFLAG-CMV-4-UBE2QL1^C88A^ and pFLAG-CMV-4 were seeded at the same density into soft agar and incubated for five weeks. SKRC 47 cells expressing UBE2QL1^C88A^ (UBE2QL1^C88A^) produced similar numbers of large (>100 μm) colonies compared with cells not expressing UBE2QL1 (EV) (*t*-test, error bars = SEM, *P* = 0.3677, *n* = 6). Below the graph are representative images of clones following 5 weeks of incubation (×100 magnification).

To determine if *UBE2QL1* suppresses growth of RCC cells we transfected wild-type *UBE2QL1* expression plasmids into the *UBE2QL1* silenced RCC cell lines SKRC 47 and SKRC 39 (data not shown) and undertook colony formation assays. Reexpression of *UBE2QL1* was associated with a significant (57.5% and 54.6%, respectively) reduction in colonies compared with those transfected with empty plasmid in both SKRC 47 and SKRC 39 cells (Fig. 3A). We then assessed the effect of reexpression of *UBE2QL1* on anchorage-independent growth in a soft agar colony formation assay. Stably transfected SKRC 47 cells expressing *UBE2QL1* or EV were compared following incubation in soft agar for five weeks. Compared with EV, there was a statistically significant 77% reduction in colony growth in *UBE2QL1* transfected cells (Fig. 3B).

### *UBE2QL1* Associated Tumorigenesis is not Associated with HIF Dysregulation

As HIF dysregulation plays a key role in *VHL*-associated renal tumorigenesis, we investigated expression of the HIF-1 and HIF-2 targets [carbonic anhydrase 9 (CA9) and cyclin D1 (CCND1), respectively] in three t(5;19)(p15.3;q12) associated renal tumors from individuals III:1 and III:2 (two oncocytomas and one chromophobe RCC). In contrast to control *VHL*-inactivated RCC, none of the tumors demonstrated upregulation of CA9 or CCND1 protein expression suggesting a HIF-independent mechanism of *UBE2QL1* tumorigenesis (Supp. Fig. S6).

### UBE2QL1 is an E2 Ubiquitin Conjugating Enzyme

**Figure 4:**
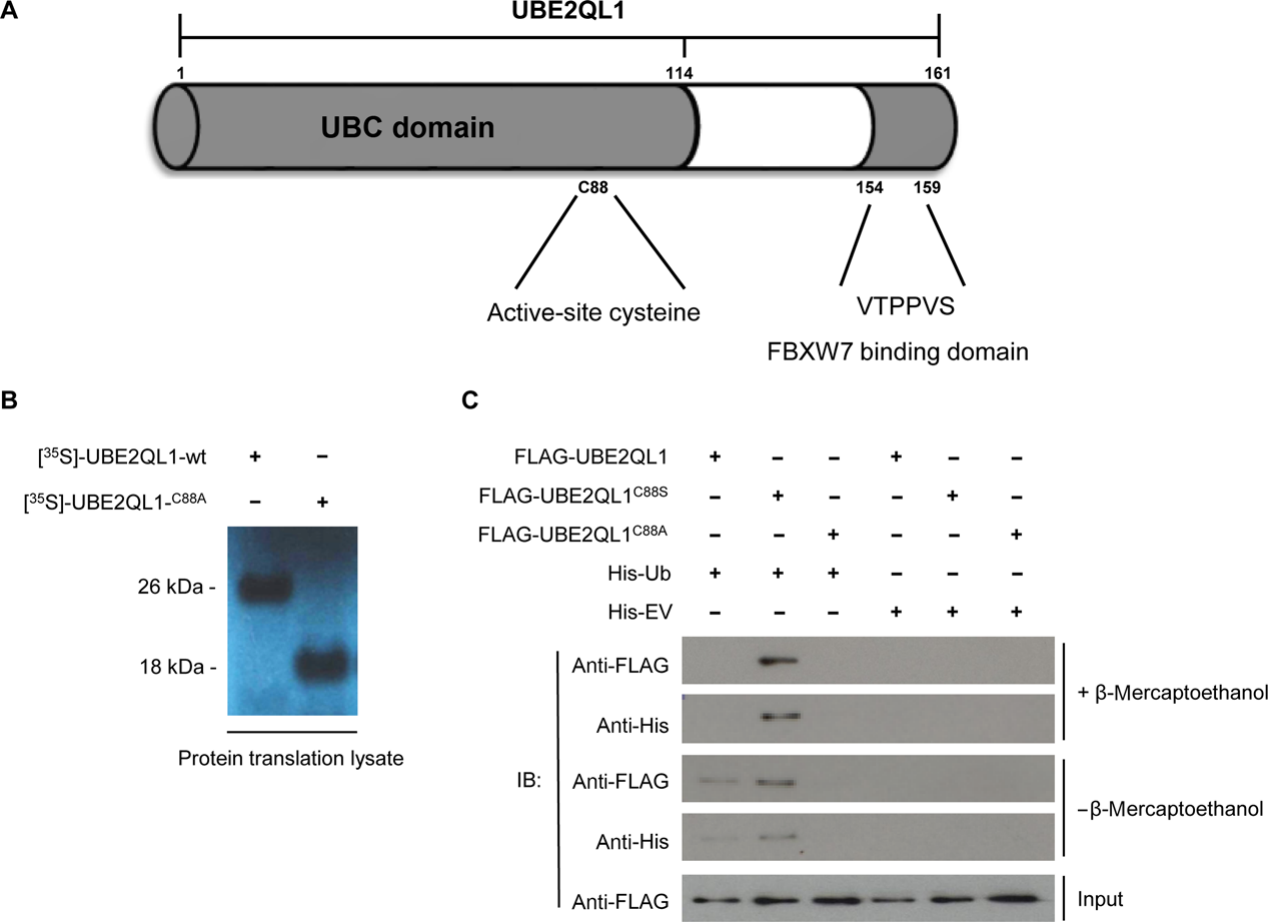
*UBE2QL1* binds monoubiquitin in vivo. A: Schematic diagram of UBE2QL1 showing the ubiquitin conjugating (Ubc) domain containing an active-site cysteine at residue 88 and a FBXW7 recognition motif (residues 154–159). B: Transcription/translation lysate system produces *wt*UBE2QL1 at ∼26 kDa and UBE2QL1^C88A^ at ∼18 kDa suggests *wt*UBE2QL1 is monoubiquitinated (ubiquitin M_r_ 8.5 kDa). C: HEK-293 cells were transfected with either FLAG-tagged UBE2QL1-wt or UBE2QL1-C88S or FLAG-UBE2QL1-C88A mutants and either His_6_-tagged ubiquitin (His-Ub) or empty vector (His-EV). His pulldown followed by immunoblot (IB) analysis demonstrated that UBE2QL1-wt is monoubiquitinated in vivo (bands at *M*_r_ of ∼26 kDa). Input levels of UBE2QL1-wt, UBE2QL1-C88S, and UBE2QL1-C88A in the cell lysate are indicated (bands at *M*_r_ of ∼18 kDa).

The amino-acid sequence of UBE2QL1 shows homology to the E2 class of ubiquitin conjugating enzymes which are characterized by a 150 residue catalytic core domain, known as the Ubc domain, containing a central active-site cysteine, which binds ubiquitin through a thiolester bond [Wenzel et al., 2010] (Fig. 4A). E2s form an important component of the ubiquitylation cascade whereby ubiquitin is initially activated by an E1 enzyme followed by transfer to an E2 and, once charged with ubiquitin, the E2 interacts with an E3 ubiquitin ligase to ubiquitylate the protein substrate with substrate fate being determined by the nature of the ubiquitin chain(s) formed [Ikeda and Dikic 2008; Pickart 2001].

We hypothesized that UBE2QL1 functions as an E2 conjugating enzyme binding ubiquitin via a thiolester bond through the cysteine residue at position 88. We initially expressed wild-type UBE2QL1, and a predicted nonfunctional p.Cys88Ala (C88A) UBE2QL1 mutant, using L-α-[^35^S]-methionine and an in vitro transcription/translation rabbit reticulocyte lysate system that supports ubiquitin conjugation. Consistent with our idea that cysteine 88 of UBE2QL1 binds ubiquitin, wild-type UBE2QL1 migrated with a band size of 26 kDa, whereas the UBE2QL1 C88A mutant migrated with a size of 18 kDa (Fig. 4B). These findings were consistent with the hypothesis that p.Cys88 binds ubiquitin and we then proceeded to investigate whether UBE2QL1 binds ubiquitin directly in vivo.

We transfected HEK-293 cells with expression plasmids encoding HIS_6_–ubiquitin and FLAG-*wt*UBE2QL1 or FLAG-UBE2QL1^C88S^ or FLAG-UBE2QL1^C88A^. The p.Cys88Ser mutant was investigated as the thiolester bond which forms between an E2 active-site cysteine and ubiquitin, while essential for catalysis, is often not able to withstand cell lysis procedures, whereas ubiquitin interaction with serine forms a more stable oxy-ester complex and is predicted to enhance ubiquitin binding [Wada et al., 2000].

Ubiquitin binding was assessed by pull-down with Dynabeads® and blotting with antibodies against the HIS_6_ and FLAG tags in both the absence and presence of β-mercaptoethanol (which reduces thiolester bonds). In the absence of β-mercaptoethanol, *wt*UBE2QL1 and UBE2QL1^C88S^ bound ubiquitin with the C88S showing enhanced avidity for ubiquitin relative to *wt*UBE2QL1 (Fig. 4C). Of note, UBE2QL1 bound monoubiquitin and did not form polyubiquitin chains. In the presence of β-mercaptoethanol, and consistent with observations for some other ubiquitin conjugating enzymes, *wt*UBE2QL1 lost its ability to bind ubiquitin, whereas the UBE2QL1^C88S^ mutant retained its ability to bind ubiquitin (Fig. 4C). These results indicate that UBE2QL1 is monoubiquitinated in vivo through the active-site cysteine, C88.

### Ub Binding is Necessary for *UBE2QL1*-Mediated RCC Cell Growth Suppression

Having demonstrated that UBE2QL1 binds monoubiquitin at its active-site cysteine, we then proceeded to determine if this binding was essential for the growth suppressive function of UBE2QL1 in the soft agar colony formation assay. Stably transfected SKRC 47 cells expressing either the nonfunctional p.Cys88Ala (C88A) UBE2QL1 mutant or EV were compared following incubation in soft agar for 5 weeks. There was no significant difference in the colony counts, indicating that the growth suppressive function of UBE2QL1 is dependent upon its ability to bind ubiquitin at the C88 position (Fig. 3C).

### UBE2QL1 Interacts with FBXW7

The interaction between E2 enzymes and E3 ligases is often weak (this enables the efficient dissociation after ubiquitin transfer of the E2 enzyme from the E3 ligase, to make way for a new E2-ubiquitin bound molecule) and many E2–E3 complex interactions are not detectable using normal protein interaction detection methods, such as yeast-2-hybrid and immunoprecipitation/mass spectrometry. Furthermore, most E3 ligases will only bind ubiquitin bound E2 enzymes [Deshaies and Joazeiro, 2009]. We did not detect a UBE2QL1 E3 ligase binding partner through conventional detection methods and so undertook an in silico search (http://elm.eu.org/) for potential binding motifs in the full length UBE2QL1 protein sequence. This revealed a consensus sequence, VTPPVS at positions 154–159, which has been proposed to act as an FBXW7 recognition motif (Fig. 4A) [Nash et al., 2001]. FBXW7 is an F-box protein that provides substrate recognition to the CUL1-SKP1-RBX1 SCF ubiquitin ligase (SCF^FBXW7^) complex [Bai et al., 1996; Feldman et al., 1997; Skowyra et al., 1997; Skowyra et al., 1999] and has previously been reported to be somatically mutated in human cancers and to be disrupted by a RCC-associated constitutional translocation [Davis and Tomlinson 2012; Kuiper et al., 2009; Tan et al., 2008; Welcker and Clurman 2008].

**Figure 5:**
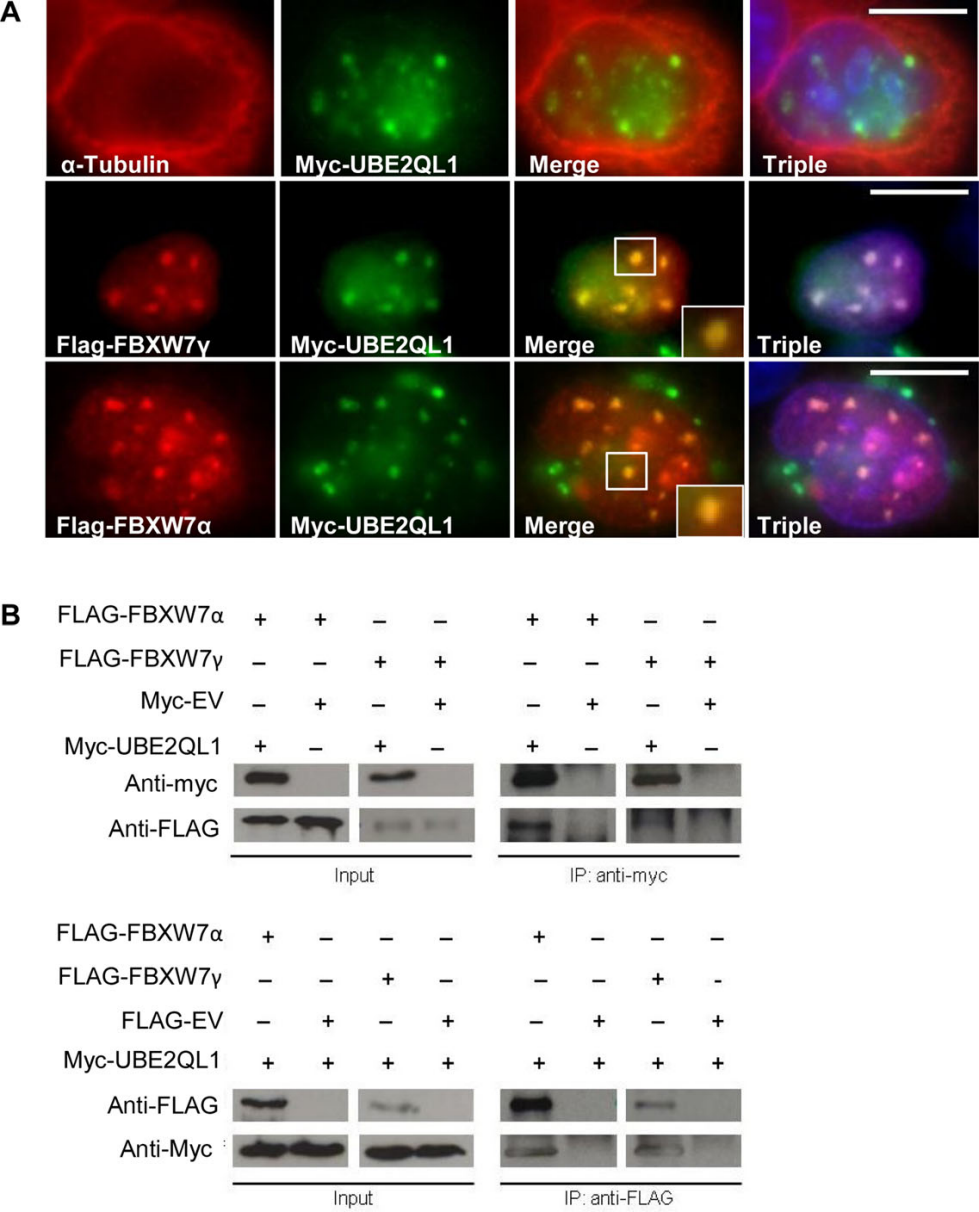
UBE2QL1 colocalizes and immunoprecipitates with FBXW7. A: HeLa cells were transfected with either myc-UBE2QL1 alone and stained with antibodies against α-tubulin (red) and myc (green), which showed a nuclear localization of UBE2QL1 (upper panel). When cotransfected with myc-UBE2QL1 and either FBXW7 γ (middle panel) or FBXW7α (lower panel) and stained with antibodies against FBXW7α or FBXW7γ (red) and myc (green) there was nuclear colocalization of UBE2QL1 with FBXW7α and FBXW7γ. Triple refers to DAPI nuclear staining (blue) and the merged images together. B: HEK-293 cells were transfected with either myc tagged to an empty vector (myc-EV) or myc-UBE2QL1 and FLAG-FBXW7 as indicated. Immunoprecipitation (IP) of myc-UBE2QL1 (upper panel) followed by immunoblot (IB) analysis with antibody against the FLAG tag identified FBXW7α and FBXW7γ as UBE2QL1 interacting proteins. The reciprocal experiment whereby IP of FLAG-FBXW7α and FLAG-FBXW7γ (lower panel) followed by IB analysis with antibody against the myc tag also identified FBXW7α and FBXW7γ as UBE2QL1 interacting proteins. Input levels of FBXW7α, FBXW7γ, and UBE2QL1 in the cell lysate are indicated.

To investigate whether UBE2QL1 and FBXW7 might interact, we investigated the intracellular localization of myc-UBE2QL1 and FLAG-α/γ-FBXW7 (there are no suitable antibodies for the endogenous proteins) in HeLa cells. Previously, the α and γ isoforms of FBXW7 were shown to be present in the nucleus [Welcker and Clurman, 2008]. We confirmed these findings (data not shown) and detected nuclear colocalization of UBE2QL1 with both the α and γ isoforms of FBXW7 (Fig. 5A). Furthermore, after transfection of HEK293 cells with FLAG-α/γ-FBXW7 and myc-UBE2QL1, immunoprecipitation and immunoblot analysis, FBXW7 and UBE2QL1 were found to coimmunoprecipitate with antibodies against either the FLAG or myc tag and immunoblotting with the reciprocal antibody (Fig. 5B).

### Effect of UBE2QL1 on Expression of FBXW7 Targets

**Figure 6:**
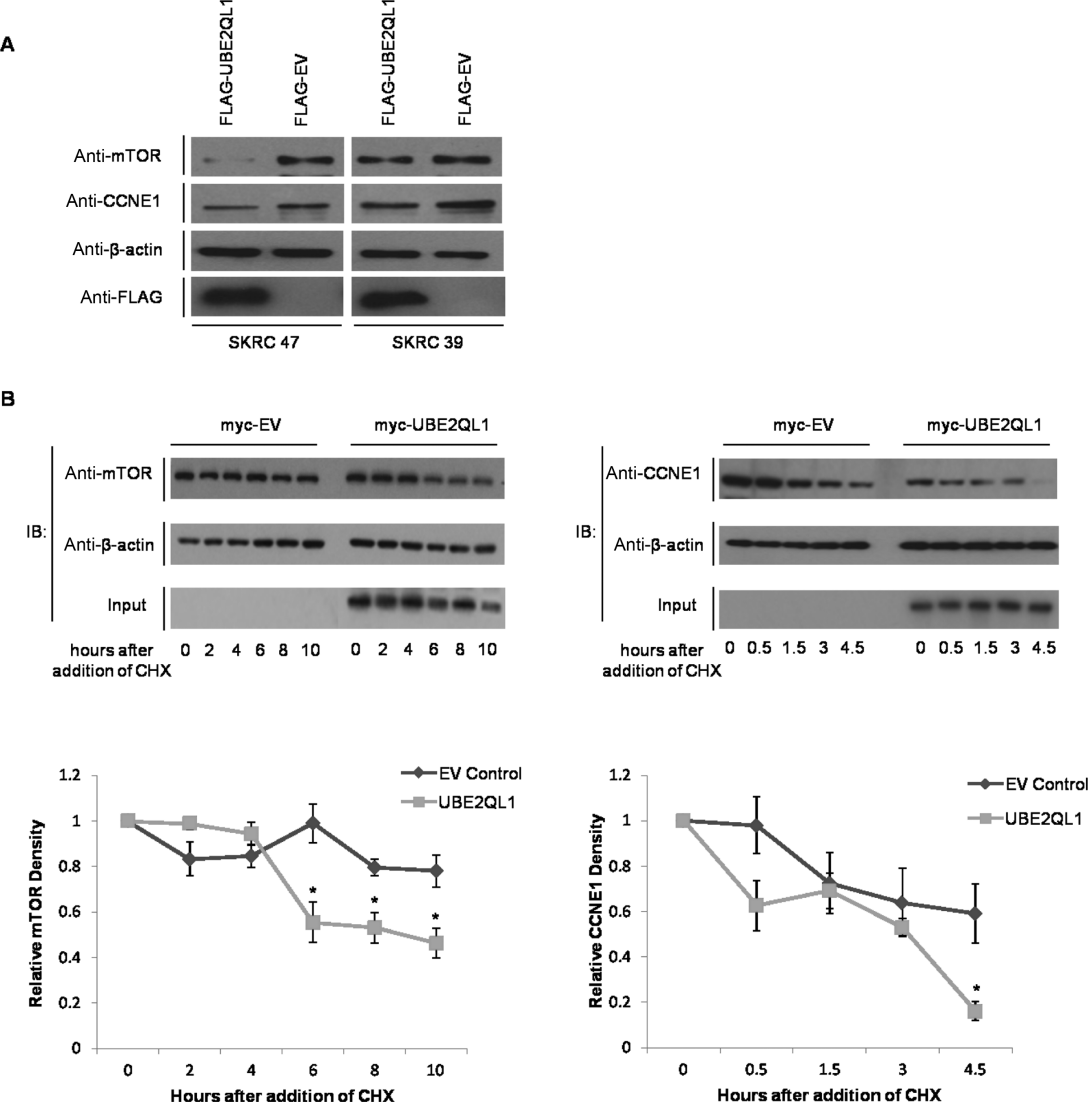
UBE2QL1 facilitates the degradation of FBXW7 targets mTOR and CCNE1. A: 10 μg of protein lysate extracted from SKRC 47 and SKRC 39 stable clones expressing pFLAG-CMV-4-*wt*UBE2QL1 (FLAG-UBE2QL1) and pFLAG-CMV-4 (FLAG-EV) were immunoblotted with antibodies against both mTOR and CCNE1 and demonstrated a reduction in their expression in UBE2QL1 expressing cells as compared with the EV control. Immunoblot controls for anti-ß-actin and anti-FLAG are also shown. B: HeLa cells were transfected with either myc tagged to an empty vector (myc-EV) or myc-UBE2QL1 as indicated. Twenty-four hours post transfection, cells were treated with 100 μg/ml cyclohexamide (CHX) and collected at the indicated times afterward. Upper panels, immunoblot analysis using antibodies against endogenous mTOR (left) and CCNE1 (right) indicated a reduction in protein levels compared with that of the housekeeping protein, β-actin. Input level of UBE2QL1 in the cell lysate is shown. Lower panels, relative densities of mTOR (left) and CCNE1 (right) to β-actin by densitometry, normalized to time point zero (unpaired *t*-test, error bars = SEM, *n* = 3, *P < 0.05).

Previous studies have reported that FBXW7 regulates expression of CyclinE1 (CCNE1) and mTOR [Koepp et al., 2001; Mao et al., 2008]. We proceeded to investigate whether UBE2QL1 status might influence CCNE1 and mTOR expression in the UBE2QL1 deficient RCC cell lines SKRC 47 and SKRC 39. Protein lysates from the UBE2QL1 or EV stably transfected SKRC 47 and SKRC 39 cells were used to compare protein expression levels of mTOR and CCNE1. Both CCNE1 and mTOR expression was reduced in UBE2QL1 expressing cells compared with EV controls (Fig. 6A). We then investigated the degradation of both FBXW7 targets in SKRC 47 cells after transient transfection with myc-UBE2QL1 or EV and addition of the protein synthesis inhibitor, cyclohexamide. A serial reduction in both mTOR and CCNE1 protein levels was detected after transfection with the myc-UBE2QL1 plasmid (as compared with cells transfected with EV) indicating that degradation of these FBXW7 targets is enhanced by UBE2QL1 (Fig. 6B). In view of the in vitro evidence that UBE2QL1 regulated CCNE1 expression, t(5;19)(p15.3;q12) associated renal tumors from individuals III:I and III:II (two oncocytomas and one chromophobe RCC) were stained for CCNE1 expression and increased expression detected (Supp. Fig. S7).

## Discussion

We identified an uncharacterized gene, *UBE2QL1*, that was disrupted by a t(5;19)(p15.3;q12) associated with a familial predisposition to RCC, and demonstrated that *UBE2QL1* has tumor suppressor activity and is inactivated in a subset of sporadic RCC by promoter region hypermethylation and/or deletions. Interestingly, although the mechanism of tumor suppression of several inherited RCC genes (e.g., *VHL*, *FH*, *FLCN*, and *SDHB*) has been linked to HIF-1/HIF-2 related pathways, we did not identify evidence of HIF target dysregulation in t(5;19)(p15.3;q12) associated renal tumors (and there was no relationship between *UBE2QL1* status and the presence or absence of a *VHL* mutation in sporadic RCC). The function of UBE2QL1 has not previously been characterized but it shows homology to the E2 class of ubiquitin conjugating enzymes and we found evidence of UBE2QL1 monoubiquitination (consistent with an E2 function). Furthermore, after identifying by computational analysis a candidate FBXW7 recognition motif in UBE2QL1 we found evidence for UBE2QL1 interacting with FBXW7 and regulating expression of the FBXW7 targets CCNE1 and mTOR. Further investigations are required to elucidate the precise function of UBE2QL1 and the relationship between UBE2QL1 growth suppression, E2 activity and FBXW7 function. Although components of E3 ubiquitin complexes (e.g., VHL and FBXW7) have been clearly implicated in tumorigenesis [Bernassola et al., 2008; Sun, 2003; Sun 2006], there is relatively little information regarding the potential role of E2 conjugating enzymes, though increased expression of some (e.g., UbcH10 and E2-EPF) has been described in some cancers [Okamoto et al., 2003; Roos et al., 2011; Seghatoleslam et al., 2012; Tedesco et al., 2007]. Based on the known tumor suppressor function of FBXW7 and the previous report of an RCC associated constitutional translocation that disrupts *FBXW7*, we suspect that UBE2QL1 inactivation compromises FBXW7 function although further studies are required to elucidate the exact mechanism. However, it is likely that further UBE2QL1 binding partners remain to be identified and their role in RCC tumorigenesis elucidated.

Previously, the FBXW7/SCF complex was shown to target mTOR for ubiquitylation and proteasomal degradation [Mao et al., 2008] and mTOR activation is common in sporadic RCC and mTOR inhibitors have shown promise in clinical trials for the treatment of metastatic RCC [Anandappa et al., 2010; Gerullis et al., 2010; Pinto Marín et al., 2012]. We note that although *VHL*-inactivated RCC are invariably clear cell, those associated with germline *FLCN* mutations (causing Birt–Hogg–Dubé syndrome) and the t(5;19)(p15.3;q12) represent a variety of histopathological subtypes indicating that UBE2QL1 dysregulation, like FLCN, can drive tumorigenesis of different RCC subtypes. Furthermore, in accord with our findings with respect to UBE2QL1, the FLCN gene product has been implicated in mTOR pathway regulation [Baba et al., 2006; Chen et al., 2008; Hasumi et al., 2009].

Recently Guo et al. (2011) undertook exome and targeted resequencing in RCC and identified twelve previously unidentified genes mutated at elevated frequencies in clear cell RCC. They highlighted the role of mutations in components of the ubiquitin-mediated proteolysis pathway (UMPP), and found an association between UMPP mutations and dysregulation of HIF-1 and HIF-2. Our findings illustrate how analysis of rare inherited forms of RCC can provide candidate TSGs that would not be detected by exome resequencing studies (as somatic inactivation of *UBE2QL1* occurred through epigenetic silencing and deletions) and note that the absence of frequent intragenic *UBE2QL1* mutations is reminiscent of *RASSF1A* which is often inactivated by methylation/allele loss in sporadic RCC [Dreijerink et al., 2001; Morrissey et al., 2001; Richter et al., 2009]. The absence of a detectable mechanism of inactivation in some tumors showing UBE2QL1 downregulation, and the finding of two distinct hits in just a minority of tumors, suggests that further alterations affecting UBE2QL1 function, either of UBE2QL1 itself, or via another component of the pathway, remain to be identified. Thus, dysregulation of FH and SDHB can contribute to HIF-1α-mediated pseudo-hypoxia driving RCC tumorigenesis through substrate inhibition of HIF-1α prolyl hydroxylation [Pollard et al., 2005] and, more recently, the identification of *TCEB1* (MIM #600788) (encoding Elongin C) mutations in sporadic RCC [Sato et al., 2013].

## Notes

Disclaimer: Supplementary materials have been peer-reviewed but not copyedited.
