# Supplementary material for: UBE2QL1 is Disrupted by a Constitutional Translocation Associated with Renal Tumor Predisposition and is a Novel Candidate Renal Tumor Suppressor Gene
Source: Hum Mutat. 2013 Oct 7;34(12):1650–61. doi: 10.1002/humu.22433 (PMC4028990; doi:10.1002/humu.22433)
Supplement: Supplementary file 1 [file humu0034-1650-sd1.pdf]

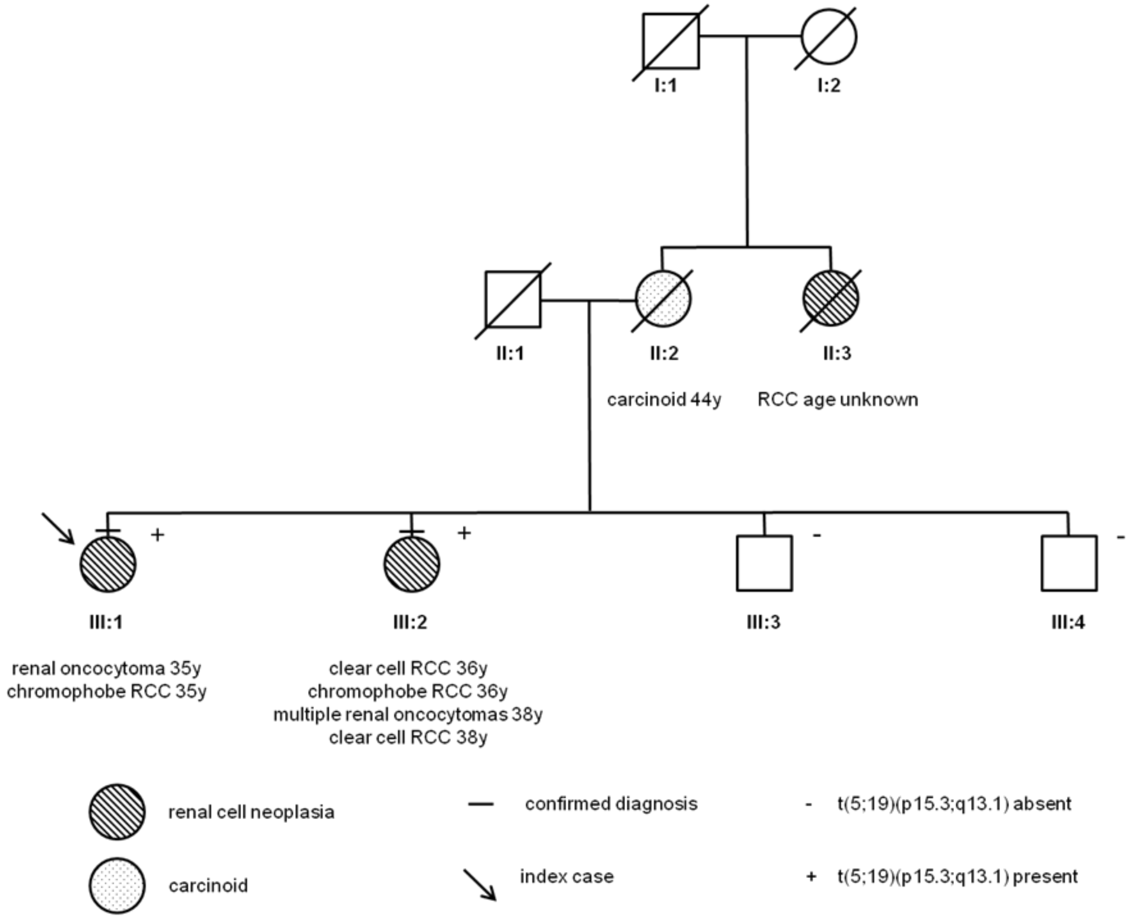

Supp. Figure S1. Pedigree of family with constitutional t(5;19)(p15.3;q13.1) and predisposition to renal cell carcinoma (RCC). Affected family members and those with the constitutional t(5;19)(p15.3;q13.1) are indicated as per the figure legend. Females are represented by a circle and males by a square. Deceased individuals are indicated by a line across the respective symbols. III:1 represents the index case.

A

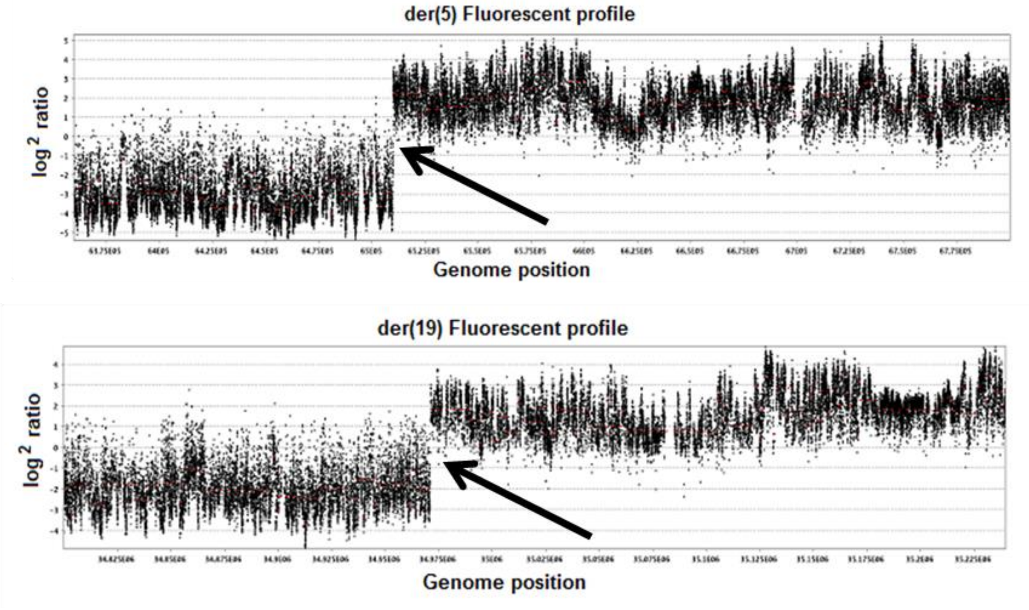

B

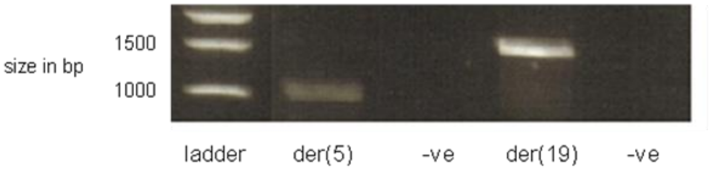

Supp. Figure S2. Fine mapping of der(5) and der(19) breakpoints. A, The fluorescent profiles of both der(5) and der(19) from the t(5;19)(p15.3;q12) are shown following hybridisation of derivative chromosomes onto a custom designed oligonucleotide array. The estimated breakpoint positions are indicated by an arrow. B, Long range PCR products following amplification across the breakpoints for the der(5) and der(19) chromosomes. The PCR products were then gel extracted and sequenced using Sanger sequencing to obtain the sequences shown in Figure 1. –ve = negative control for the addition of template.

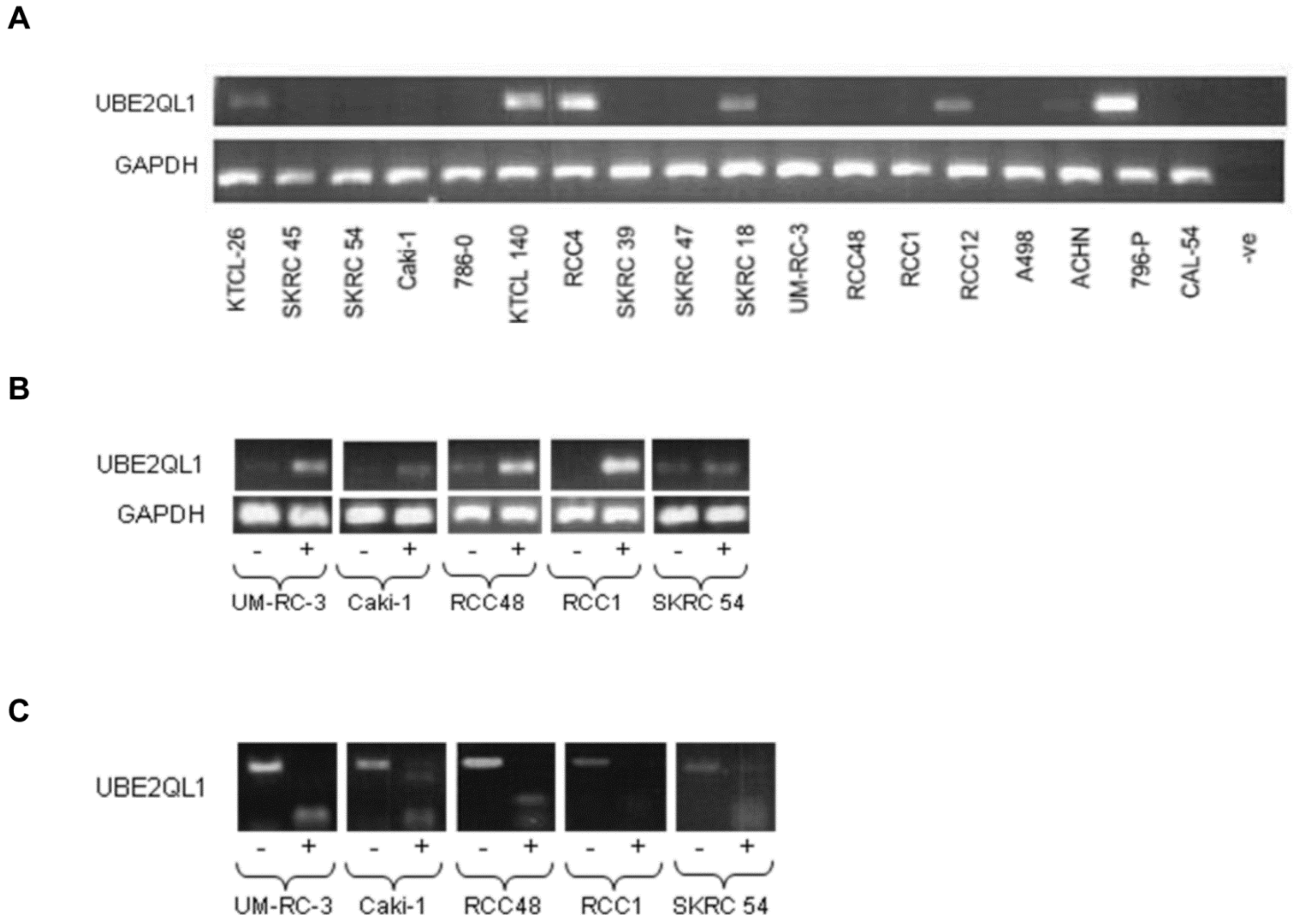

Supp. Figure S3. Expression and methylation analyses of *UBE2QL1* in RCC cell lines. A, Reverse transcriptase PCR showing loss of *UBE2QL1* expression in 11/18 RCC cell lines as compared with *GAPDH* expression. '–ve' = negative control for the addition of template. B, Reverse transcriptase PCR showing increased expression of *UBE2QL1* following growth in the demethylating agent 5-Aza-2'-deoxycytidine (5-Aza) in five RCC cell lines suggesting promoter hypermethylation as a means of inactivation. '-' grown in absence of 5-Aza, '+' grown in presence of 5-Aza. C, BstU1 digest analysis following PCR amplification of the *UBE2QL1* CpG island using bisulphite modified DNA as template (COBRA) in the five RCC cell lines showing increased *UBE2QL1* expression after being grown in 5-Aza. All five cell lines showed digestion products indicating *UBE2QL1* promoter region hypermethylation. '-' no BstU1 added, '+' BstU1 added.

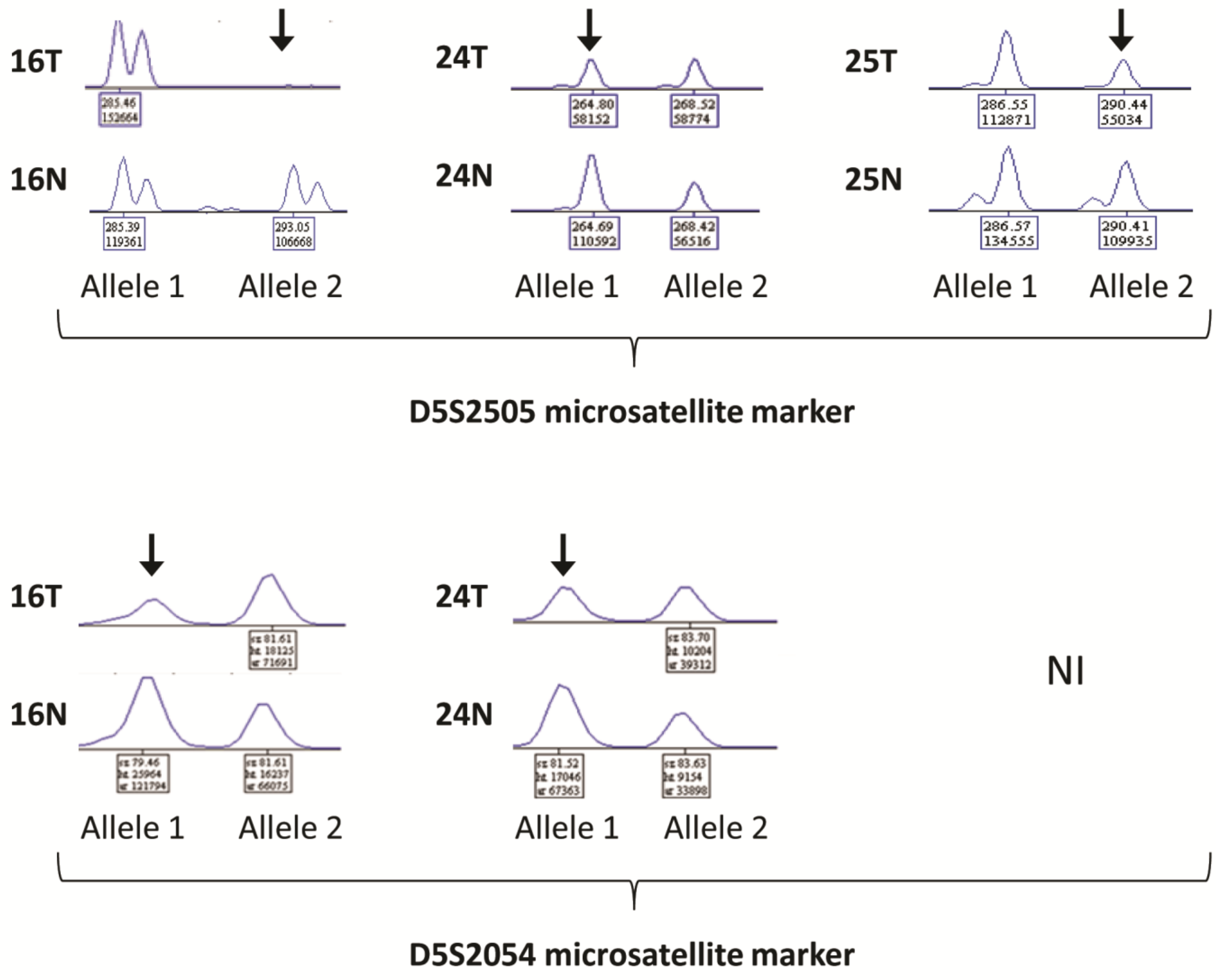

Supp. Figure S4. Loss of heterozygosity (LoH) of 5p13.3 in sporadic RCC tumours. Traces for three tumours (16T, 24T and 25T) showing LoH of the microsatellite marker D5S2505 and two tumours (16T and 24T) showing LoH of the microsatellite marker D5S2054. Both markers reside in chromosome band 5p15.3. T = tumour, N = normal kidney tissue from the same individual, NI = non-informative. LoH was deemed to be present where the peak height ratio (peak height of allele 1 / peak height of allele 2) of tumour to normal kidney tissue was ≤0.5.

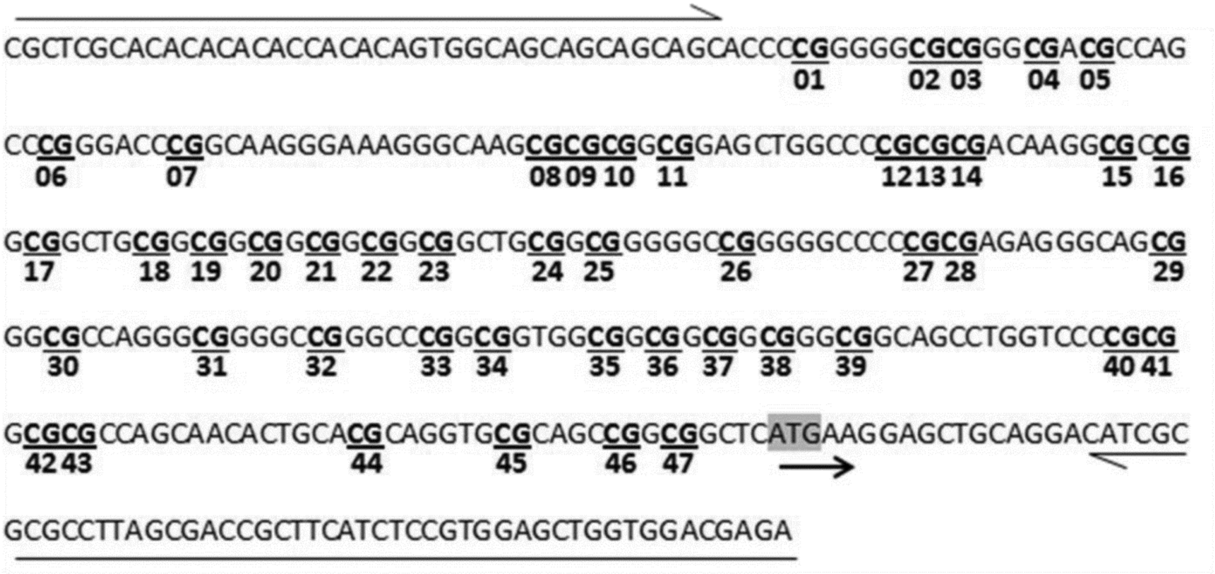

Supp. Figure S5. CpG island region of *UBE2QL1* [NM_001145161, chr5:6448710-6449006 (hg19)]. The CpG island was identified using http://cpgislands.usc.edu/ and the criteria of Takai and Jones (2002). CpG dinucleotides analysed are numbered 1–47 and primer sequences used for nested COmbined Bisulphite Restriction Analysis (COBRA) shown with the half arrows. The translational start site is highlighted and the direction of translation indicated by a full arrow.

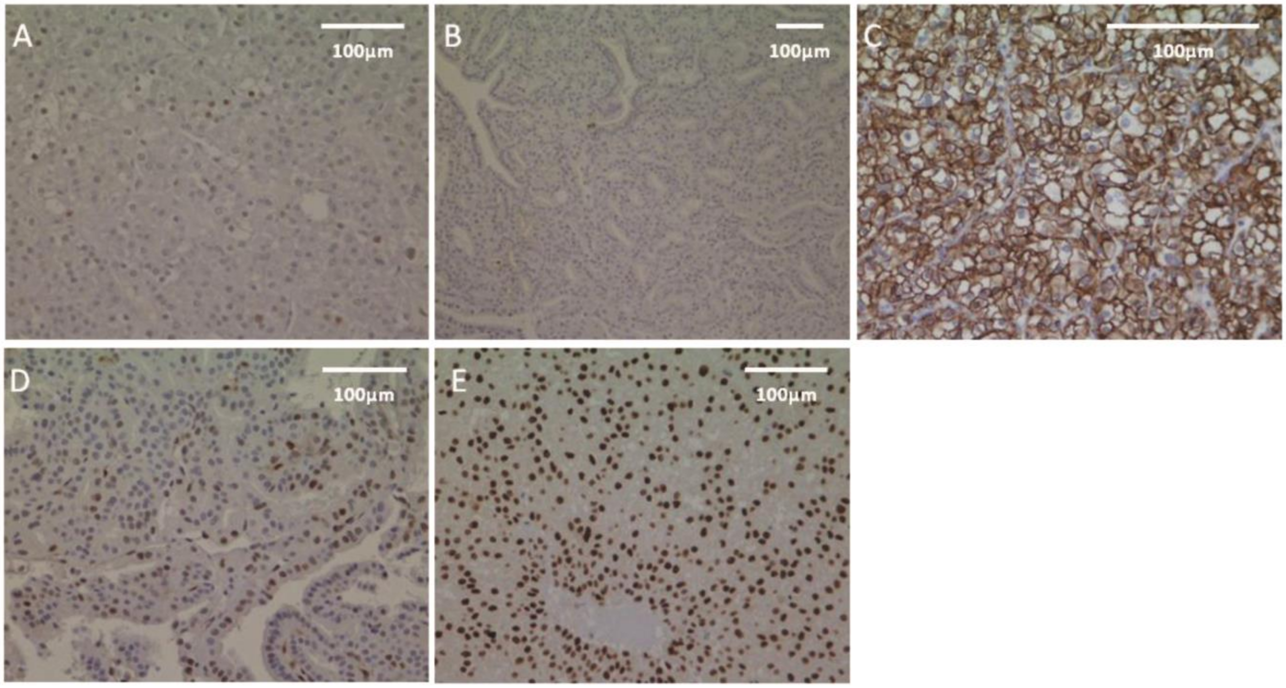

Supp. Figure S6. Immunohistochemical analysis of hypoxia inducible factor (HIF) targets in t(5;19)(p15.3;q13.1) associated renal tumours**.** A, Carbonic anhydrase 9 (CA9) immunohistochemistry (IHC) in a t(5;19)(p15.3;q13.1) associated oncocytoma showing no evidence of up-regulation. B, CA9 IHC in a t(5;19)(p15.3;q13.1) associated chromophobe renal tumour showing no evidence of up-regulation. C, CA9 IHC in a sporadic clear cell RCC showing up-regulation for comparison with (A) and (B). D, CyclinD1 (CCND1) IHC in the t(5;19)(p15.3;q13.1) associated chromophobe RCC showing no evidence of up-regulation. E, CCND1 IHC in a sporadic chromophobe RCC showing up-regulation for comparison with (D).

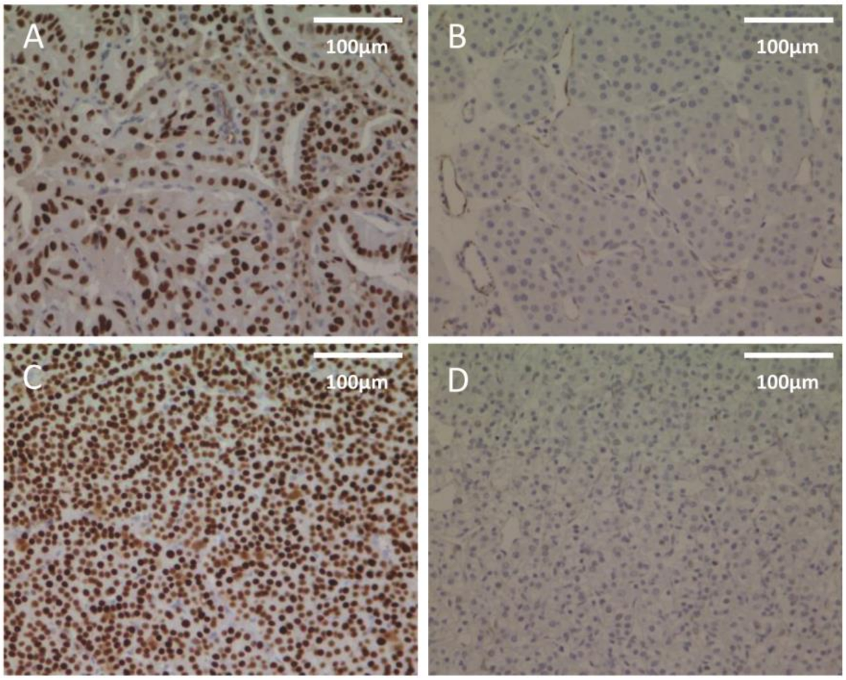

Supp. Figure S7. Immunohistochemical analysis of cyclin E1 (CCNE1) in t(5;19)(p15.3;q13.1) associated renal tumours. A, CCNE1 immunohistochemistry (IHC) in a t(5;19)(p15.3;q13.1) associated renal oncocytoma showing evidence of up-regulation. B, CCNE1 IHC in a sporadic renal oncocytoma showing no evidence of up-regulation for comparison with (A). C, CCNE1 IHC in a t(5;19)(p15.3;q13.1) associated chromophobe RCC showing evidence of up-regulation. D, CCNE1 IHC in a sporadic clear cell RCC showing no evidence of up-regulation for comparison with (A) and (C). All images are at x10 magnification.
